# Pairwise relatedness testing in the context of inbreeding: expectation and variance of the likelihood ratio

**DOI:** 10.1007/s00414-020-02426-6

**Published:** 2020-09-28

**Authors:** Hilde Kjelgaard Brustad, Magnus Dehli Vigeland, Thore Egeland

**Affiliations:** 1 Norwegian University of Life Sciences, 1432 Aas, Norway; 2 Department of Medical Genetics, University of Oslo, PB 4956 Nydalen, 0424 Oslo, Norway

**Keywords:** Kinship analysis, Inbred founders, IBD triangle, Jacquard coefficients, Likelihood ratios

## Abstract

In this paper we investigate various effects of inbreeding on the likelihood ratio (LR) in forensic kinship testing. The basic setup of such testing involves formulating two competing hypotheses, in the form of pedigrees, describing the relationship between the individuals. The likelihood of each hypothesis is computed given the available genetic data, and a conclusion is reached if the ratio of these exceeds some pre-determined threshold. An important aspect of this approach is that the hypotheses are usually not exhaustive: The *true* relationship may differ from both of the stated pedigrees. It is well known that this may introduce bias in the test results. Previous work has established formulas for the expected value and variance of the LR, given the two competing hypotheses and the true relationship. However, the proposed method only handles cases without inbreeding. In this paper we extend these results to all possible pairwise relationships. The key ingredient is formulating the hypotheses in terms of Jacquard coefficients instead of the more restricted Cotterman coefficients. While the latter describe the relatedness between outbred individuals, the more general Jacquard coefficients allow any level of inbreeding. Our approach also enables scrutiny of another frequently overlooked source of LR bias, namely background inbreeding. This ubiquitous phenomenon is usually ignored in forensic kinship computations, due to lack of adequate methods and software. By leveraging recent work on pedigrees with inbred founders, we show how background inbreeding can be modeled as a continuous variable, providing easy-to-interpret results in specific cases. For example, we show that if true siblings are subjected to a test for parent-offspring, moderate levels of background inbreeding are expected to inflate the LR by more than 50%.

## Introduction

The conventional approach to forensic kinship testing includes formulating two hypotheses and calculating a likelihood ratio (LR) based on genetic data from genotyped individuals. Practice differs between countries and laboratories, but typically the LR or some version of it is included when the case is reported. The conclusion based on the LR may be flawed when the true pedigree connecting the individuals of interest differs from the pedigrees considered by the hypotheses. As an example, consider a standard paternity case, where the prosecution asserts that a certain man is the father of a child, while the defense claims that the man and the child are unrelated. The truth, on the other hand, may be that the man is the child’s uncle. A special case of incorrect hypotheses occurs when inbreeding is not accounted for. For example, if the alleged father is inbred, and this is ignored when formulating the hypotheses, this may significantly bias the LR. One aim of this paper is to investigate and quantify this effect.

Slooten and Egeland derived explicit equations for the expected value and variance of the LR [1]. They also extended this to cases where the true relationship differs from those stated in the hypotheses [2]. However, in both of these works only non-inbred individuals were considered. An important contribution of this paper is the extension of these results to general pairwise relationships. In particular, we show that exact expressions for the expected value and variance of the LR can be obtained also in cases with inbreeding. The expressions are in general more involved than in the non-inbred case, and not as easy to interpret. However, we derive interesting and practical results in important special cases.

A parametric approach to modeling background inbreeding in kinship testing was recently introduced [3], employing the concept of inbred founders [4]. To exemplify, consider a pair of paternal half siblings, whose father is assigned an inbreeding coefficient *f*. As *f* increases from 0 to 1, the relationship between the half siblings becomes genetically indistinguishable from that between parent and child. We extend the theoretical framework of [1, 2] to pedigrees with inbred founders. As a result, the impact of background inbreeding on the expectation and variance of the LR can be studied based on exact expressions. In cases where the amount of inbreeding is unknown, we can still provide guidance on the expected values for the LR. Our approach conveniently allows a continuous range of possible true alternatives rather than a discrete set of specific alternatives. To arrive at explicit results of practical interest, we restrict attention to pairwise relationships. Furthermore, as in the work of Slooten and Egeland, we ignore mutations, dropouts, and silent alleles and we assume Hardy-Weinberg Equilibrium (HWE). However, we explain how deviation from HWE can be modeled by the so called theta (*𝜃*) correction.

R scripts and functions used to obtain numerical results in this paper are gathered in a R library (see the “R implementation” section). Pedigree likelihoods and marker simulations are performed with the forrel package [3].

This paper is organized in the following manner: After establishing some terminology and notation we review the main results of [2] regarding the expected value and variance of the LR for non-inbred pairs of individuals. We then proceed to extend these results to general pairwise relationships, including relationships in pedigrees with background inbreeding. Several worked examples follow, including a simulation study comparing our formulas with real-life results. Finally, we discuss some consequences of this work and how it relates to other aspects of forensic genetics.

## Definitions and notation

A central concept for measuring genetic relatedness is that of *identity by descent* (IBD). Two alleles are said to be IBD relative to a given pedigree if they are identical by state and originate from the same ancestral allele within the pedigree [5].

### Coefficients of inbreeding and kinship

The *coefficient of inbreeding* *f*, introduced by Wright [6], is the probability that an individual is autozygous at a given autosomal locus, i.e., that the two homologous alleles are IBD. This is the same as the *kinship coefficientφ* between the parents of the same individual, defined as the probability that a random allele from the mother is IBD to a random allele from the father at the same locus.

Founders of a pedigree are conventionally assumed to be unrelated and non-inbred. Following [3] we relax the second assumption, allowing an arbitrary inbreeding coefficient *f* to be assigned to any founder individual. For a given pedigree with *N* founders, we denote the set of founder inbreeding coefficients by $\boldsymbol {f} = (f_{1}, f_{2},\dots ,f_{N})$.

Background inbreeding in human populations is normally low, but may exceed 5*%* in certain cases [7, 8]. In forensic case work inbreeding is common, ranging from consanguineous marriages between cousins, *f* = 1/16 or lower, to incestuous relationships between siblings or parent-child, both with *f* = 1/4. In breeding applications values closer to 1 may occur.

### Jacquard coefficients and likelihood of a pedigree

The kinship coefficient is a coarse measure of relatedness; for instance, it has the same value for a parent-child relationship as for full siblings. A more refined measure is given by the nine *Jacquard coefficients* [9] $\boldsymbol {{\varDelta }}= ({\varDelta }_{1}, \dotsc , {\varDelta }_{9})$, also called the *condensed* *identity* *coefficients*. These are the expected relative frequencies of the

**Fig. 1:**
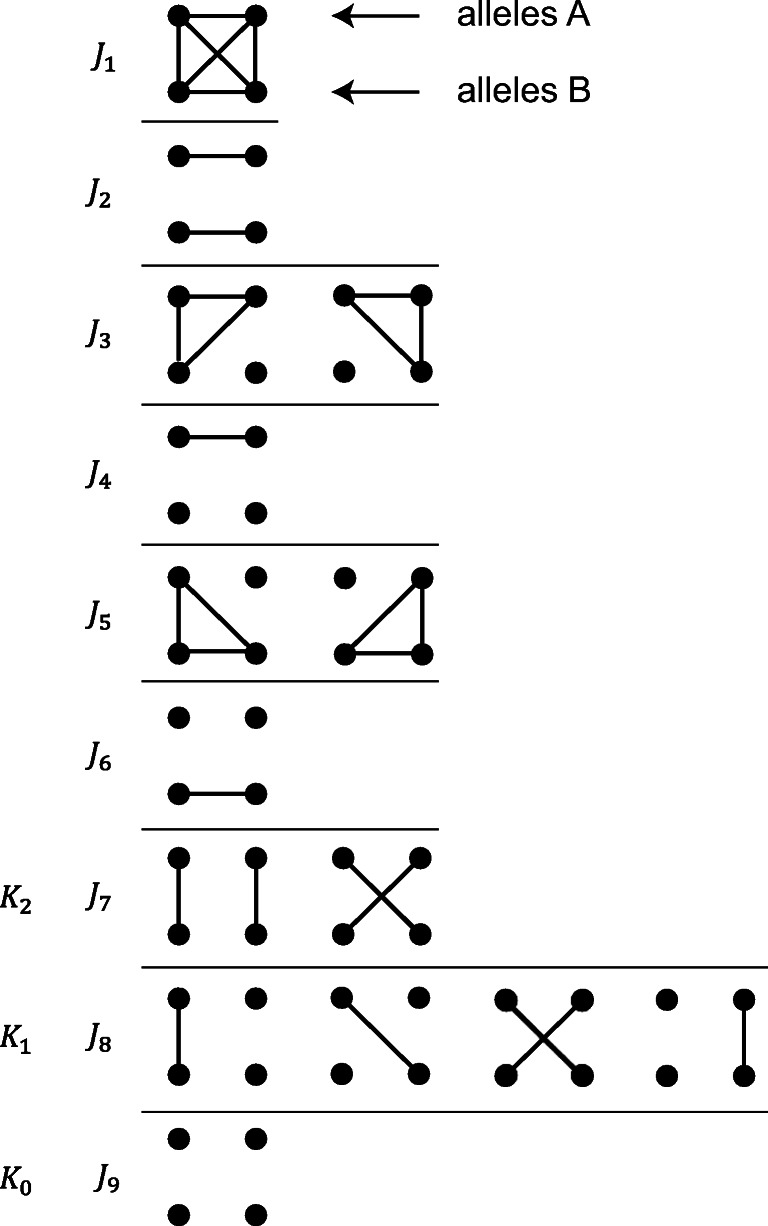
The Jacquard states $J_{1},\dots , J_{9}$ representing all possible IBD patterns among the four alleles of two individuals at an autosomal locus. Each row of dots represents the two alleles of an individual. Connected dots indicate IBD. The states *J*_9_, *J*_8_, and *J*_7_ do not involve inbreeding and are sometimes denoted *K*_0_, *K*_1_, and *K*_2_

*Jacquard* *states* $J_{1}, \dotsc , J_{9}$ are depicted in Fig. 1. Alleles within each individual are unordered, and hence, several IBD configurations can correspond to the same Jacquard state. Furthermore, **Δ** is related to *φ* through

$$ \varphi = {\varDelta}_{1}+\frac{1}{2}({\varDelta}_{3}+{\varDelta}_{5}+{\varDelta}_{7})+\frac{1}{4}{\varDelta}_{8}. $$

The likelihood of two individuals being related according to **Δ**, given their genotypes *G* = (*g*_1_,*g*_2_) at a marker may be expressed by conditioning on the Jacquard state:

1 $$ L(\boldsymbol{{\varDelta}} \mid G)=\sum\limits_{i=1}^{9} {\varDelta}_{i}P(G\mid J_{i}).  $$

The conditional probabilities *P*(*G*∣*J*_*i*_) are listed in Table 1. These probabilities are found by direct calculations; for instance, *P*((*aa*,*aa*)∣*J*_1_) = *p*_*a*_ since *J*_1_ dictates that all four alleles are IBD.

**Table 1:** The conditional probability *P*(*G*∣*J*_*i*_) of a pair of genotypes *G* = (*g*_1_,*g*_2_), given a Jacquard state *J*_*i*_

*G*	*J*_1_	*J*_2_	*J*_3_	*J*_4_	*J*_5_	*J*_6_	*J*_7_	*J*_8_	*J*_9_
(*aa*,*aa*)	*p*_*a*_	${p_{a}^{2}}$	${p_{a}^{2}}$	${p_{a}^{3}}$	${p_{a}^{2}}$	${p_{a}^{3}}$	${p_{a}^{2}}$	${p_{a}^{3}}$	${p_{a}^{4}}$
(*aa*,*bb*)	0	*p*_*a*_*p*_*b*_	0	$p_{a}{p_{b}^{2}}$	0	${p_{a}^{2}}p_{b}$	0	0	${p_{a}^{2}}{p_{b}^{2}}$
(*aa*,*ab*)	0	0	*p*_*a*_*p*_*b*_	$2{p_{a}^{2}}p_{b}$	0	0	0	${p_{a}^{2}}p_{b} $	$2{p_{a}^{3}}p_{b}$
(*aa*,*bc*)	0	0	0	2*p*_*a*_*p*_*b*_*p*_*c*_	0	0	0	0	$2{p_{a}^{2}}p_{b}p_{c}$
(*ab*,*aa*)	0	0	0	0	*p*_*a*_*p*_*b*_	$2{p_{a}^{2}}p_{b}$	0	${p_{a}^{2}}p_{b}$	$2{p_{a}^{3}}p_{b}$
(*bc*,*aa*)	0	0	0	0	0	2*p*_*a*_*p*_*b*_*p*_*c*_	0	0	$2{p_{a}^{2}}p_{b}p_{c}$
(*ab*,*ab*)	0	0	0	0	0	0	2*p*_*a*_*p*_*b*_	*p*_*a*_*p*_*b*_(*p*_*a*_ + *p*_*b*_)	$ 4{p_{a}^{2}}{p_{b}^{2}}$
(*ab*,*ac*)	0	0	0	0	0	0	0	*p*_*a*_*p*_*b*_*p*_*c*_	$4{p_{a}^{2}}p_{b}p_{c}$
(*ab*,*cd*)	0	0	0	0	0	0	0	0	4*p*_*a*_*p*_*b*_*p*_*c*_*p*_*d*_

The symbols *a*, *b*, *c*, and *d* represent different alleles, with population frequencies *p*_*a*_, *p*_*b*_, *p*_*c*_, and *p*_*d*_ respectively

### IBD coefficients and inbred founders

**Fig. 2:**
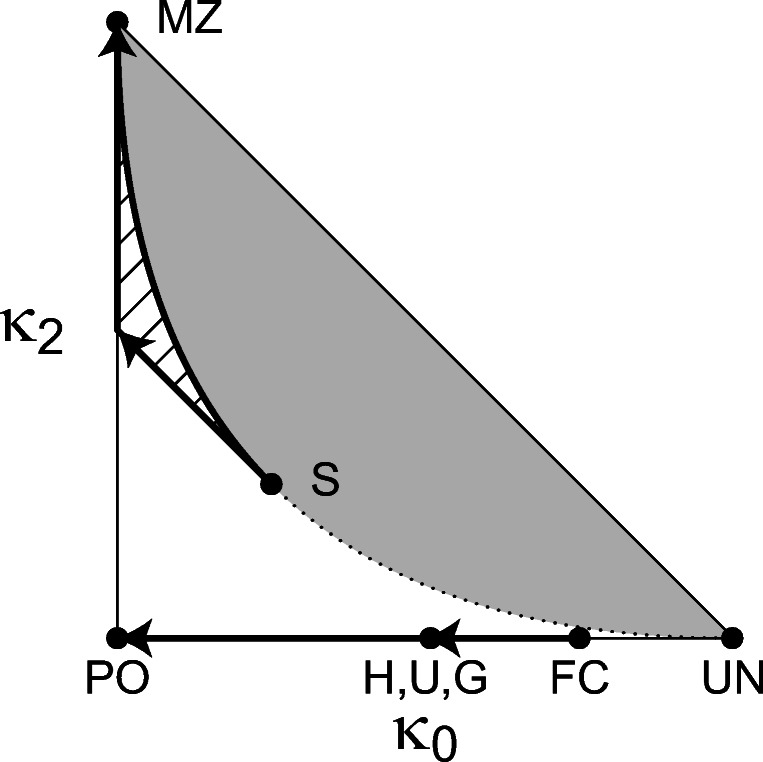
The IBD triangle with location of some common relationships. The gray area is inadmissable. The arrows illustrate the effect of founder inbreeding in the cases given in Table 2. PO, parent-child; MZ, monozygotic twins; S, siblings; H, half siblings; U, avuncular; G, grandparent grandchild; FC, first cousins; UN, unrelated

For two non-inbred individuals, the first six Jacquard coefficients are zero, and *Δ*_9_, *Δ*_8_, and *Δ*_7_ reduce to the IBD coefficients ***κ*** = (*κ*_0_,*κ*_1_,*κ*_2_) introduced by Cotterman [10]. They give the probabilities that, at a given autosomal locus, the individuals share zero-, one-, and two-allele IBD, respectively. Note that *κ*_0_ + *κ*_1_ + *κ*_2_ = 1, so ***κ*** can be represented in a two-dimensional triangle with axes *κ*_0_ and *κ*_2_. Thompson [11] showed that the IBD coefficients are restricted to ${\kappa _{1}^{2}}\geq 4\kappa _{0}\kappa _{2}$. This gives rise to an inadmissible region for the parameters, in gray in Fig. 2.

Although the IBD coefficients are only defined for non-inbred individuals, other members of the pedigree can be inbred. For example, a pair of half siblings remain outbred even if their shared parent is inbred. However, this inbreeding will affect the relatedness coefficients. Table 2 lists the kinship and the IBD coefficients for some common relationships, as functions of the founder inbreeding. The effects are visualized in Fig. 2. In the half sibling example, the genetic relationship approaches that of parent-child, as the founder inbreeding increases towards 1. Similarly, the IBD coefficients of full siblings with inbred parents may fall anywhere in the lightly shaded region towards the point of monozygotic twins.

**Table 2:** Relatedness coefficients as functions of founder inbreeding, in a selection of common relationships

	Relationship	*φ*	*φ*(***f***)	***κ***	***κ***(***f***)
	S	$\frac {1}{4}$	$\frac {1}{4}(1+\frac {f_{1}+f_{2}}{2})$	$(\frac {1}{4},\frac {1}{2},\frac {1}{4})$	$\kappa _{0}(f_{1},f_{2})=\frac {1}{4}(1-f_{1})(1-f_{2})$
					$\kappa _{1}(f_{1},f_{2})=\frac {1}{2}(1-f_{1}f_{2})$
					$\kappa _{2}(f_{1},f_{2})=\frac {1}{4}(1+f_{1})(1+f_{2}))$
	H	$\frac {1}{8}$	$\frac {1}{8}(1+f)$	$(\frac {1}{2},\frac {1}{2},0)$	$\kappa _{0}(f)=\frac {1}{2}(1-f)$
					$\kappa _{1}(f)=\frac {1}{2}(1+f)$
					*κ*_2_(*f*) = 0
	U	$\frac {1}{8}$	$\frac {1}{8}(1+\frac {f_{1}+f_{2}}{2})$	$(\frac {1}{2},\frac {1}{2},0)$	$\kappa _{0}(f_{1},f_{2})=\frac {1}{2}(1-\frac {f_{1}+f_{2}}{2})$
					$\kappa _{1}(f_{1},f_{2})=\frac {1}{2}(1+\frac {f_{1}+f_{2}}{2})$
					*κ*_2_(*f*_1_,*f*_2_) = 0
	FC	$\frac {1}{16}$	$\frac {1}{16}(1+\frac {f_{1}+f_{2}}{2})$	$(\frac {3}{4},\frac {1}{4},0)$	$\kappa _{0}(f_{1},f_{2})=\frac {1}{4}(3-\frac {f_{1}+f_{2}}{2})$
					$\kappa _{1}(f_{1},f_{2})=\frac {1}{4}(1+\frac {f_{1}+f_{2}}{2})$
					*κ*_2_(*f*_1_,*f*_2_) = 0

## Review of previous results

We next review the main results of [2] relevant for our work. In particular we restate the explicit formulas for the expectation and variance of the LR in the case of non-inbred individuals.

### The likelihood ratio as a random variable

We consider a kinship test involving genetic data from two non-inbred individuals. Two hypotheses *H*_*P*_ and *H*_*D*_ about the relationship are to be compared using the LR. For our purposes, each hypothesis corresponds to a point in the IBD triangle, denoted by ***κ***_*P*_ and ***κ***_*D*_ respectively. However, the evidence may be generated from another pedigree, corresponding to a third point ***κ***_*T*_. We therefore have the following setup, comprising the competing hypotheses and the true relationship:

$$ \begin{array}{@{}rcl@{}} &&H_{P}: \quad\boldsymbol{\kappa}=\boldsymbol{\kappa}_{P}=({\kappa^{P}_{0}},{\kappa^{P}_{1}},{\kappa^{P}_{2}})\\ &&H_{D}: \quad\boldsymbol{\kappa}=\boldsymbol{\kappa}_{D}=({\kappa^{D}_{0}},{\kappa^{D}_{1}},{\kappa^{D}_{2}}) =(1,0,0)\\&& Truth: \quad\boldsymbol{\kappa} =\boldsymbol{\kappa}_{T}=({\kappa^{T}_{0}},{\kappa^{T}_{1}},{\kappa^{T}_{2}}). \end{array} $$

Reflecting standard practice, we will always use *unrelatedness* as the defense hypothesis, i.e., ***κ***_*D*_ = (1,0,0). It should be noted, however, that this is not a theoretical requirement for the methods presented here.

The concept of the likelihood ratio as a random variable was discussed by Slooten and Egeland [1]. We review the basics here, presented in a slightly simpler notation sufficient for our purposes.

Denote by *K*_*i*_, *i* = 0,1,2, the event that the individuals share exactly *i* alleles IBD. As shown in Fig. 1, *K*_0_, *K*_1_, and *K*_2_ are identical to the Jacquard states *J*_9_, *J*_8_, and *J*_7_ respectively. For fixed ***κ***_*P*_ the likelihood ratio for a given pair of genotypes *G* = (*g*_1_,*g*_2_) can be written as

2 $$ \begin{array}{@{}rcl@{}} LR(G) = \frac{P(G | H_{P})}{P(G | H_{D})} &=& \frac{P(G | \boldsymbol{\kappa}_{P})}{P(G | \boldsymbol{\kappa}_{D})}\\ &=& \sum\limits_{i=0}^{2} {\kappa_{i}^{P}} \frac{P(G | K_{i})}{P(G | K_{0})}. \end{array} $$

Note that the final transition was obtained by applying (1) in both the numerator and denominator. The probabilities *P*(*G*|*K*_*i*_) are given in Table 1.

Now, viewing the genotypes as a random variable $\mathcal {G}$, we define the random variable ${\mathscr{L}}\mathcal {R} = LR(\mathcal {G})$. Note that the distribution of $\mathcal {G}$ is completely determined by ***κ***_*T*_ (assuming HWE), hence the distribution of ${\mathscr{L}}\mathcal {R}$ is determined by ***κ***_*P*_ and ***κ***_*T*_. If these parameters are clear from the context, we will suppress them in our notation; otherwise, we write ${\mathscr{L}}\mathcal {R}_{\boldsymbol {\kappa }_{P}, \boldsymbol {\kappa }_{T}}$. In the special case when *H*_*P*_ equals the truth, i.e., ***κ***_*P*_ = ***κ***_*T*_, we may simplify ${\mathscr{L}}\mathcal {R}_{\boldsymbol {\kappa }_{P},\boldsymbol {\kappa }_{T}}$ to ${\mathscr{L}}\mathcal {R}_{\boldsymbol {\kappa }_{P}}$.

Throughout, we assume the following condition to hold

3 $$ P(G \mid H_{P})>0 \Rightarrow P(G \mid H_{D})>0.  $$

In the present context, it means that all DNA profiles that can occur under *H*_*P*_, can also occur under *H*_*D*_. In our examples *H*_*D*_ specifies unrelated individuals, and then (3) holds. The condition also holds for mutation models provided all elements of the mutation matrix are positive. We do not model mutations in the work presented here, as practical exact expression are then no longer available. However, the implementation allows for general mutation models. Without (3), likelihood ratios could be infinite, i.e., not defined.

### Expected likelihood ratio

The expectation of ${\mathscr{L}}\mathcal {R}$ may be found by summing over all possible genotypes *G* in the standard way:

4 $$ E(\mathcal{L}\mathcal{R}) = \sum\limits_{G} P(G) LR(G), $$

where $P(G) = P(G | \boldsymbol {\kappa }_{T}) = {\sum }_{i} {\kappa _{i}^{T}} P(G | K_{i})$. An exact expression for $E({\mathscr{L}}\mathcal {R})$ when ***κ***_*P*_ = ***κ***_*T*_ was first derived in [1] and extended in [2] to apply when ***κ***_*P*_≠***κ***_*T*_. For the latter situation it was shown that, for a single marker with *L* alleles,

5 $$ E(\mathcal{L}\mathcal{R})= \boldsymbol{\kappa}_{P} \cdot A_{0} \cdot (\boldsymbol{\kappa}_{T})^{t},  $$

where *t* denotes the vector transpose, and

6 $$ A_{0}= \begin{pmatrix} 1 & 1 & 1 \\ 1 & \frac{L+3}{4} & \frac{L+1}{2} \\ 1 & \frac{L+1}{2}& \frac{L(L+1)}{2} \end{pmatrix}.  $$

Importantly, the expected value depends only on the number of alleles, not on the allele frequencies. Furthermore, the expectation is symmetric in ***κ***_*P*_ and ***κ***_*T*_, so that

7 $$ E(\mathcal{L}\mathcal{R}_{\boldsymbol{\kappa}_{P},\boldsymbol{\kappa}_{T}})=E(\mathcal{L}\mathcal{R}_{\boldsymbol{\kappa}_{T},\boldsymbol{\kappa}_{P}}).  $$

### Variance of the likelihood ratio

To derive the variance of ${\mathscr{L}}\mathcal {R}$ we apply the general formula $\text {var}(\mathcal {X}) = E(\mathcal {X}^{2}) - E(\mathcal {X})^{2}$. Since the last term follows from Eq. 5, all that remains is to find the first term. Some notation is needed:

$$ \begin{array}{ll} s_{1} &= \frac{1}{16}\sum\limits_{a<b} \left( \frac{p_{a}}{p_{b}}+\frac{p_{b}}{p_{a}} \right), \\ s_{2} &= \sum\limits_{a<b}\frac{1}{2p_{a}p_{b}}, \\ s_{3} &= \sum\limits_{a}\frac{1}{p_{a}}, \\ s_{4} &= \frac{1}{4}\sum\limits_{a<b}\left( \frac{1}{p_{b}}+\frac{1}{p_{a}}\right), \\ s_{5} &= \sum\limits_{a}\frac{1}{{p_{a}^{2}}}. \end{array} $$

Furthermore, supplementing the matrix *A*_0_ given in Eq. 6, we define matrices *A*_1_ and *A*_2_ by

8 $$ \begin{array}{l} A_{1}= \begin{pmatrix} 1 & \frac{L+3}{4} & \frac{L+1}{2} \\ \frac{L+3}{4} & \frac{5L+3}{8}+s_{1} &\frac{L(L+7)}{8}+2s_{1}\\ \frac{L+1}{2} & \frac{L(L+7)}{8}+2s_{1}&s_{3}+s_{4} \end{pmatrix} \end{array}  $$

9 $$ A_{2}= \begin{pmatrix} 1 &\frac{L+1}{2} &\frac{L(L+1)}{2} \\ \frac{L+1}{2} & \frac{L(L+7)}{8}+2s_{1} &s_{3}+s_{4}\\ \frac{L (L+1)}{2} &s_{3}+s_{4}&s_{2}+s_{5} \end{pmatrix}  $$

It was shown in [2] that

$$ E(\mathcal{L}\mathcal{R}^{2}) = \sum\limits_{i=0}^{2} {\kappa^{P}_{i}} \boldsymbol{\kappa}_{P} A_{i} (\boldsymbol{\kappa}_{T})^{t}; $$

hence, the complete variance expression becomes

10 $$ \begin{array}{@{}rcl@{}} &&\text{var}(\mathcal{L}\mathcal{R}) = \\ &&\sum\limits_{i=0}^{2} {\kappa^{P}_{i}} \boldsymbol{\kappa}_{P} A_{i} (\boldsymbol{\kappa}_{T})^{t} - \left( \boldsymbol{\kappa}_{P} A_{0} (\boldsymbol{\kappa}_{T})^{t}\right)^{2}. \end{array} $$

Contrary to the expected LR, the variance of the LR depends on the allele frequencies.

### Example: paternity testing

This example serves as an illustration of the above described expected LR and the corresponding hypotheses. Consider a paternity case, where a man is claimed to be the father of a child (*H*_*P*_). The truth is that a brother of the alleged father is the true father of the child. The hypotheses and the true relatedness are in terms of the IBD coefficients given as

11 $$ \begin{array}{ll} H_{P}: \quad\boldsymbol{\kappa}=\boldsymbol{\kappa}_{P}&=(0,1,0)\\ H_{D}: \quad\boldsymbol{\kappa}=\boldsymbol{\kappa}_{D}&=(1,0,0)\\ Truth: \quad\boldsymbol{\kappa} =\boldsymbol{\kappa}_{T}&=(\frac{1}{2},\frac{1}{2},0). \end{array} $$

**Fig. 3:**
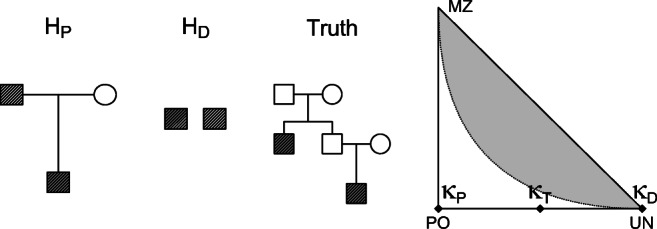
Pedigrees and location of IBD coefficients ***κ***_*P*_, ***κ***_*D*_, and ***κ***_*T*_ for a paternity case when the true relationship is avuncular

Figure 3 illustrates the hypotheses in terms of pedigrees, and as points in the IBD triangle. Equation (5), with IBD coefficients as in Eq. 11, simplifies to

12 $$ E(\mathcal{L}\mathcal{R})=\frac{L+7}{8}.  $$

The variance of ${\mathscr{L}}\mathcal {R}$ becomes

$$ \begin{array}{l} \text{var}(\mathcal{L}\mathcal{R})=\frac{7L+9}{16}+\frac{s_{1}}{2}-{\left( \frac{L+7}{8}\right) }^{2}. \end{array} $$

In the special case *L* = 2, and allele frequencies *q* and 1 − *q*, the variance expression reduces to

$$ \text{var}(\mathcal{L}\mathcal{R}) = \frac{11}{64}+\frac{1}{32}\frac{{(1-q)}^{2}+ q^{2}}{q(1-q)}. $$

This expression is minimal when *q* = 0.5 and becomes infinitely large when *q* or 1 − *q* approaches 0. If no assumption is made for *L*, but all alleles are assumed equally frequent, the variance reduces to

13 $$ \text{var}(\mathcal{L}\mathcal{R}) = \frac{L(L+12)}{64} - \frac{13}{64}.  $$

Table 3 exemplifies these formulas for various realistic values of *L*, and compares the results with the corresponding values if *H*_*P*_ was true.

**Table 3:** Expectation and variance of ${\mathscr{L}}\mathcal {R}$ in the paternity example of Fig. 3, for loci with 2, 10, and 50 alleles

Truth	***κ***_*P*_	***κ***_*T*_	E[LR]	*L* = 2	*L* = 10	*L* = 50
PO	(0, 1, 0)	(0, 1, 0)	$\frac {L+3}{4}$	1.250 (0.188)	3.250 (1.686)	13.250 (9.188)
U	(0, 1, 0)	$(\frac {1}{2},\frac {1}{2},0)$	$\frac {L+7}{8}$	1.125 (0.234)	2.125 (3.234)	7.125 (48.230)

The variances are computed assuming uniform allele frequencies. The bottom row (*U*) shows the values when the true pedigree is uncle-nephew, as analyzed in the main text. For comparison, the top row shows the corresponding numbers when *H*_*P*_ is true

## Likelihood ratio for general pairwise relationships

In this section we extend the results reviewed above to relationships between any pairs of individuals. In particular we now allow inbreeding. For this to work we must pass from the IBD coefficients to the full set of Jacquard coefficients. For details regarding derivations of the results (see the Appendix).

### Expected likelihood ratio

We use the same setup for kinship testing as introduced previously, but in order to allow general inbreeding, we now formulate our hypotheses using Jacquard coefficients,

$$ \begin{array}{ll} H_{P}:  \boldsymbol{{\varDelta}}=\boldsymbol{{\varDelta}}_{P}&=({{\varDelta}^{P}_{1}},\ldots,{{\varDelta}^{P}_{9}})\\ H_{D}:  \boldsymbol{{\varDelta}}=\boldsymbol{{\varDelta}}_{D}&=({{\varDelta}^{D}_{1}},\ldots,{{\varDelta}^{D}_{9}}) =(0,\ldots,0,1)\\ Truth:  \boldsymbol{{\varDelta}} = \boldsymbol{{\varDelta}}_{T}&=({{\varDelta}^{T}_{1}},\ldots,{{\varDelta}^{T}_{9}}). \end{array} $$

Note that the defense hypothesis still corresponds to unrelatedness. We are interested in the likelihood ratio comparing *H*_*P*_ with *H*_*D*_ when the genotypes are generated by a pedigree with the Jacquard coefficients **Δ**_*T*_. Equation (1) implies that

14 $$ \begin{array}{ll} LR(G) &= \frac{P(G | \boldsymbol{{\varDelta}}_{P})}{P(G | \boldsymbol{{\varDelta}}_{D})} \\ &= \sum\limits_{i=1}^{9} {{\varDelta}_{i}^{P}} \frac{P(G | J_{i})}{P(G | J_{9})}. \end{array} $$

As shown in the Appendix, the expected LR is

15 $$ E(\mathcal{L}\mathcal{R}_{\boldsymbol{{\varDelta}}_{P},\boldsymbol{{\varDelta}}_{T}}) = \boldsymbol{{\varDelta}}_{P}B_{9}(\boldsymbol{{\varDelta}}_{T})^{t},  $$

where *B*_9_ is the symmetric 9 × 9 matrix given in Table 4, whose elements are $E({\mathscr{L}}\mathcal {R}_{J_{i}, J_{j}})$, for 1 ≤ *i*,*j* ≤ 9. As opposed to the non-inbred case, we see that the expected value in general depends on the allele frequencies.

**Table 4:** Elements of the symmetric matrix *B*_9_, given as $E({\mathscr{L}}\mathcal {R}_{J_{i},J_{j}})$

	*J*_1_	*J*_2_	*J*_3_	*J*_4_	*J*_5_	*J*_6_	*J*_7_	*J*_8_	*J*_9_
*J*_1_	$\sum {\frac {1}{{p_{a}^{2}}}}$	$\sum {\frac {1}{p_{a}}}$	$\sum {\frac {1}{p_{a}}}$	*L*	$\sum {\frac {1}{p_{a}}}$	*L*	$\sum {\frac {1}{p_{a}}}$	*L*	1
*J*_2_		*L*^2^	*L*	*L*	*L*	*L*	*L*	1	1
*J*_3_			$\frac {1}{2}(L + \sum {\frac {1}{p_{a}}})$	*L*	*L*	1	*L*	$\frac {L+1}{2}$	1
*J*_4_				*L*	1	1	1	1	1
*J*_5_					$\frac {1}{2}(L + \sum {\frac {1}{p_{a}}})$	*L*	*L*	$\frac {L+1}{2}$	1
*J*_6_						*L*	1	1	1
*J*_7_							$\frac {L(L+1)}{2} $	$\frac {L+1}{2}$	1
*J*_8_								$\frac {L+4}{3}$	1
*J*_9_									1

Each row represents *J*_*i*_, a Jacquard state assumed by *H*_*P*_, while each column presents *J*_*j*_, the true Jacquard state

### Variance of the likelihood ratio

In the Appendix matrices $B_{1}, \dots , B_{9}$ are defined and it is shown that

16 $$ E(\mathcal{L}\mathcal{R}^{2}) = \sum\limits_{i=1}^{9} {{\varDelta}^{P}_{i}} \boldsymbol{{\varDelta}}_{P} B_{i} (\boldsymbol{{\varDelta}}_{T})^{t}. $$

From this we obtain the variance formula

17 $$ \begin{array}{@{}rcl@{}} &&\text{var}(\mathcal{L}\mathcal{R}) = \\ &&\sum\limits_{i=1}^{9} {{\varDelta}^{P}_{i}} \boldsymbol{{\varDelta}}_{P} B_{i} (\boldsymbol{{\varDelta}}_{T})^{t} - \left( \boldsymbol{{\varDelta}}_{P}B_{9}(\boldsymbol{{\varDelta}}_{T})^{t}\right)^{2}. \end{array} $$

### Pairwise relationships with inbred founders

As previously explained, a set of inbreeding coefficients ***f*** can be assigned the founders of a pedigree to model background inbreeding. The Jacquard coefficients of any pair of pedigree members are then functions of ***f***. It follows that the formulas for expectation and variance of ${\mathscr{L}}\mathcal {R}$ involving such pedigrees remain as in Eqs. 15 and 17, except that the parameters **Δ**_*P*_ and **Δ**_*T*_ must be updated.

Specifically, let ***f***_*P*_ be a vector of founder inbreeding coefficients in the pedigree assumed by *H*_*P*_, and ***f***_*T*_ similarly in the true pedigree. The expectation and variance of ${\mathscr{L}}\mathcal {R}$ in this situation are then given by

$$ E(\mathcal{L}\mathcal{R}_{\boldsymbol{{\varDelta}}_{P} (\boldsymbol{f}_{P}),\boldsymbol{{\varDelta}}_{T}(\boldsymbol{f}_{T})})=\boldsymbol{{\varDelta}}_{P}(\boldsymbol{f}_{P})B_{9}(\boldsymbol{{\varDelta}}_{T}(\boldsymbol{f}_{T}))^{t} $$

and

$$ \begin{array}{@{}rcl@{}} &&\text{var}(\mathcal{L}\mathcal{R}_{\boldsymbol{{\varDelta}}_{P}(\boldsymbol{f}_{P}),\boldsymbol{{\varDelta}}_{T}(\boldsymbol{f}_{T})})\\ &=&\sum\limits_{i=1}^{9} {{\varDelta}^{P}_{i}}(\boldsymbol{f}_{P}) \boldsymbol{{\varDelta}}_{P}(\boldsymbol{f}_{P}) B_{i} (\boldsymbol{{\varDelta}}_{T}(\boldsymbol{f}_{T}))^{t} \\ &&- \left( \boldsymbol{{\varDelta}}_{P}(\boldsymbol{f}_{P})B_{9}(\boldsymbol{{\varDelta}}_{T}(\boldsymbol{f}_{T}))^{t}\right)^{2}. \end{array} $$

Note that the matrices *B*_*i*_ only depend on *L* and the allele frequencies, and therefore are unchanged by founder inbreeding.

#### *Remark 1*

It should be emphasized that the formulas (15) and (17) are needed only when at least one of the tested individuals are inbred in some of the involved pedigrees. If both are non-inbred, the simpler expressions (5) and (10) using IBD coefficients suffice. Importantly, this remains true if *other* members of the pedigree are inbred, as long as this does not lead to inbreeding in the tested individuals. In particular, founder inbreeding may be accounted for in Eqs. 5 and 10 simply by replacing ***κ***_*P*_ and ***κ***_*T*_ by ***κ***_*P*_(***f***_*P*_) and ***κ***_*T*_(***f***_*T*_) respectively.

### Founder inbreeding and *𝜃* correction

The conventional approach to background relatedness in forensics is the so called *𝜃* correction [12]. In an inbred population, the composition of genotypes do not follow the Hardy-Weinberg principle, implying that the frequencies given in Table 1 no longer hold. The following approach compensates for this by adjusting the allele frequencies. Without loss of generality we can assume that alleles observed are sampled sequentially. The probability that allele *i* is sampled as the *j* th allele is given by the *sampling formula*

18 $$  p_{i}^{\prime}=\frac{b_{j}\theta+\bar{\theta}p_{i}}{1+(j-2)\theta}, $$

where $\bar {\theta }=1-\theta $ and *b*_*j*_ denotes the number of alleles of type *i* among the *j* − 1 previously sampled. Note that for pairwise cases, the likelihood can be written

19 $$ L(\boldsymbol{{\varDelta}}(\boldsymbol{f}) \mid G, \theta)=\sum\limits_{i=1}^{9} {\varDelta}_{i}(\boldsymbol{f})P(G\mid J_{i}, \theta),  $$

where *P*(*G*∣*J*_*i*_,*𝜃*) is calculated using Eq. 18. The matrices *B*_1_,...,*B*_9_ then change with *𝜃*, modifying the expectation and variance of the LR. This emphasises a fundamental difference between founder inbreeding and *𝜃* correction: ***f*** modifies the relationship itself, while *𝜃* only impacts the genotype probabilities.

### Example: *𝜃* correction and founder inbreeding in a paternity case

This example compares *𝜃* correction to founder inbreeding. Consider first the hypothesis *H*_*D*_: A and B are unrelated. Assume both individuals are homozygous a/a. Equation (18) gives the likelihood

$$ L_{\theta}(H_{D}) = p_{a}(\theta+\bar{\theta}p_{a}) \frac{2\theta+\bar{\theta} p_{a}}{1+\theta} \frac{3\theta+\bar{\theta} p_{a}}{1+2\theta}. $$

If rather than using *𝜃* correction, we assign an inbreeding coefficient *f* to A, the likelihood becomes

$$ L_{f}(H_{D}) = (fp_{a}+(1-f){p_{a}^{2}}){p_{a}^{2}}. $$

Consider next the hypothesis *H*_*P*1_: A is the father of B. Equation (18) now gives

$$ L_{\theta}(H_{P1}) = p_{a}(\theta+\bar{\theta}p_{a})\frac{2\theta+\bar{\theta} p_{a}}{1+\theta} $$

and so the LR with *𝜃* correction is

$$ LR_{\theta} = \frac{L_{\theta}(H_{P1})}{L_{\theta}(H_{D})} = \frac{1+2\theta}{3\theta+\bar{\theta} p_{a}}. $$

The inbreeding coefficient approach gives

$$ L_{f}(H_{P1}) = (fp_{a}+(1-f){p_{a}^{2}})p_{a} $$

and *LR*_*f*_ = 1/*p*_*a*_. Note that the LR does not depend on *f* and that this is true for all genotype combinations for A and B. The LRs for other genotype combinations for A and B with *𝜃* correction are given in Table 10.8 in [13].

To illustrate (19) consider the hypothesis *H*_*P*2_: A and B are paternal half siblings whose father is inbred. Table 2 then gives ${\varDelta }_{8} =\frac 12(1+f)$ and ${\varDelta }_{9} = \frac 12(1-f)$, and by Eqs. 18 and 19 we may write down the likelihood for any genotype combinations. For instance, when A is homozygous a/a and B homozygous b/b the likelihood is

$$ L(f, \theta)= \frac12 (1-f)p_{a}(\theta+\bar{\theta}p_{a}) \frac{\bar{\theta}p_{b}}{1+\theta} \frac{\theta+\bar{\theta}p_{b}}{1+2\theta}. $$

The LR comparing *H*_*P*2_ with A and B being unrelated becomes $\frac 12(1-f)$. If A and B share alleles, the LR will depend also on *𝜃*.

## R implementation

Utilities to perform the computations in this paper are provided in a R library named InbredLR, available from the first author, building on several packages in the *ped suite*, notably pedprobr and forrel [3]. The core of InbredLR are functions that compute the expectation and variance of the likelihood ratio for pairwise relationships. The user can specify the parameters (***κ***, ***f*** or **Δ**) or specify the pedigrees, possibly with inbred founders. A function for simulating marker data to estimate the distribution of LR is also provided, as well as a function for visualizing pedigrees *H*_*P*_ and *H*_*D*_ and the true pedigree and location of the corresponding IBD coefficients in the IBD triangle.

## Results

### Paternity case for siblings with inbred founders

**Fig. 4:**
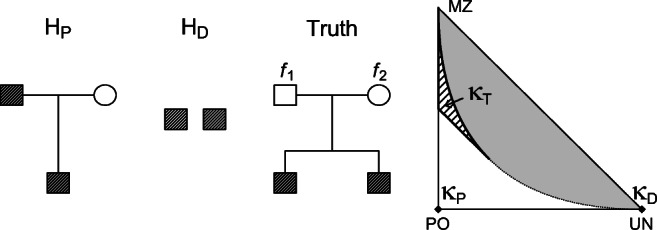
Hypotheses involved in “Paternity case for siblings with inbred founders” and the location of the corresponding IBD coefficients ***κ***_*P*_, ***κ***_*D*_, and ***κ***_*T*_ in the IBD triangle

Consider two individuals who claim to be related as parent and offspring. Their true relationship is siblings and their parents coefficients of inbreeding are ***f***_*T*_ = (*f*_1_,*f*_2_). Figure 4 shows the case. This example can be relevant for family reunion cases, where a parent-child relationship would give right to residence permit, whereas a sibling relationship would not. In [14] such a case is considered. *H*_*P*_ and *H*_*D*_ and their true relationship are in terms of the IBD coefficients given as

20 $$ \begin{array}{ll} H_{P}: \qquad&\boldsymbol{\kappa}=\boldsymbol{\kappa}_{P}=(0,1,0)\\ H_{D}: \qquad&\boldsymbol{\kappa}=\boldsymbol{\kappa}_{D}=(1,0,0)\\ Truth: \qquad&\boldsymbol{\kappa} = \boldsymbol{\kappa}_{T}(\boldsymbol{f}_{T}), \end{array}  $$

where ***κ***_*T*_(***f***_*T*_) = ***κ***_*T*_(*f*_1_,*f*_2_) are as in the first row of Table 2. Keeping in mind Remark 1, we apply (5) to find the expected LR:

21 $$ E(\mathcal{L}\mathcal{R}) = \frac{L-1}{8}(f_{1}+f_{2})+ \frac{L+3}{4}.  $$

**Fig. 5:**
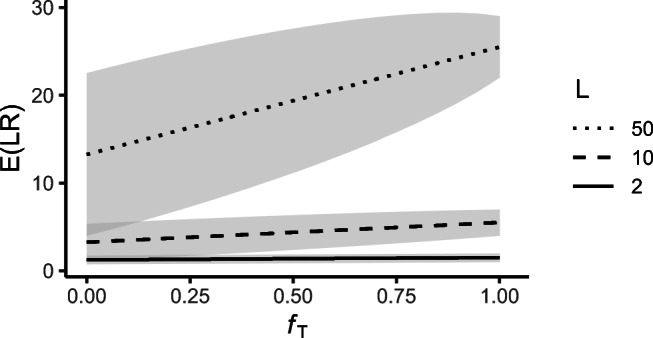
$E({\mathscr{L}}\mathcal {R})$ as function of background inbreeding level *f*_*T*_ (assuming *f*_1_ = *f*_2_), for *L* = 2, 10, and 50 alleles, for the paternity case in Fig. 4. The shaded area shows one standard deviation below and above $E({\mathscr{L}}\mathcal {R})$, for uniform allele frequencies

Figure 5 plots $E({\mathscr{L}}\mathcal {R})$ as a function of the inbreeding level (assuming *f*_1_ = *f*_2_), for a single locus with *L* = 2, 10 and 50 alleles.

Without founder inbreeding, $E({\mathscr{L}}\mathcal {R})=(L+3)/4$. Interestingly, this is the same as the expectation if *H*_*P*_ was true, i.e., if the two individuals were in fact father and son (see first row of Table 3). The variance of ${\mathscr{L}}\mathcal {R}$ differs between the two cases, however (not shown here).

As the background inbreeding of the true sibling pedigree increases, $E({\mathscr{L}}\mathcal {R})$ increases. The expected LR of the paternity case (and hence the trust in *H*_*P*_) is therefore higher if the true relatedness is siblings with background inbreeding, rather than the tested parent-child relationship. The variance of ${\mathscr{L}}\mathcal {R}$ decreases moderately for increasing founder inbreeding. For increasing number of alleles *L*, the slope of the expected LR increases.

The following calculation gives a simple approximation of the inflation in the expected LR caused by background inbreeding. Suppose *f*_1_ = *f*_2_ = *f*, and write (21) as *μ*_0_ + *μ*_*f*_, where $\mu _{0} = \frac 14(L+3)$ is the expected LR without founder inbreeding, and $\mu _{f} = \frac 14(L-1) f$ is the expected contribution caused by founder inbreeding. Note that $\mu _{0} + \mu _{f} = (1 + \frac {\mu _{f}}{\mu _{0}})\mu _{0}$, and that for *L* ≥ 5 we have $\frac {\mu _{f}}{\mu _{0}} = \frac {L-1}{L+3}f \geq \frac 12 f$. This implies that with *N* independent markers, the total LR has expectation

$$ [(1 + \tfrac{\mu_{f}}{\mu_{0}})\mu_{0}]^{N} \geq (1+ \tfrac12 f)^{N} {\mu_{0}^{N}} \geq (1 + \tfrac12fN) {\mu_{0}^{N}}. $$

This means that a background inbreeding level *f* will inflate the expected LR by at least $\frac 12 f N$. For example, if *N* = 20 and *f* = 0.05, the inflation rate is greater than 50*%*.

### Siblings and half siblings with founder inbreeding

Distinguishing between siblings and half siblings can be difficult based on unlinked markers. Mayor and Balding address the problem in [15], with focus on the number of loci needed. If the shared parent of the half siblings has inbreeding coefficient *f*_*T*_ > 0, the problem becomes even more interesting.

**Fig. 6:**
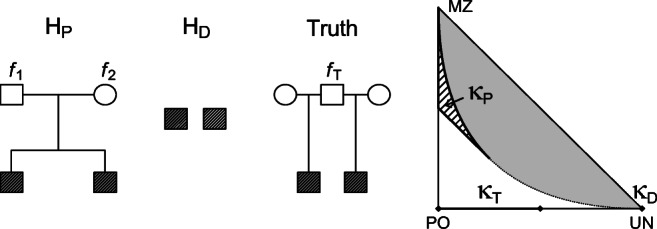
The hypotheses involved in “Siblings and half siblings with founder inbreeding” and the location of the corresponding IBD coefficients ***κ***_*P*_, ***κ***_*D*_, and ***κ***_*T*_ in the IBD triangle

Consider the situation shown in Fig. 6. The hypotheses are

22 $$  \begin{array}{ll} H_{P}: \qquad&\boldsymbol{\kappa} = \boldsymbol{\kappa}_{P}(\boldsymbol{f}_{P}) \\ H_{D}: \qquad&\boldsymbol{\kappa} = \boldsymbol{\kappa}_{D} = (1,0,0)\\ Truth: \qquad&\boldsymbol{\kappa} = \boldsymbol{\kappa}_{T}(f_{T}), \end{array} $$

where ***f***_*P*_ = (*f*_1_,*f*_2_) are the parental inbreeding coefficients in the *H*_*P*_ pedigree and ***κ***_*P*_(***f***_*P*_) and ***κ***_*T*_(*f*_*T*_) are as in the first and second rows of Table 2, respectively. This setup facilitates for modeling background inbreeding in both the true pedigree and in *H*_*P*_. Equation (5) gives

23 $$ \begin{array}{@{}rcl@{}} E(\mathcal{L}\mathcal{R}) &=&\frac{L-1}{8}\left( \frac{(f_{1}+f_{2})(f_{T}+1)}{2}+f_{T}\right)\\ &&+\frac{L+7}{8}. \end{array} $$

**Fig. 7:**
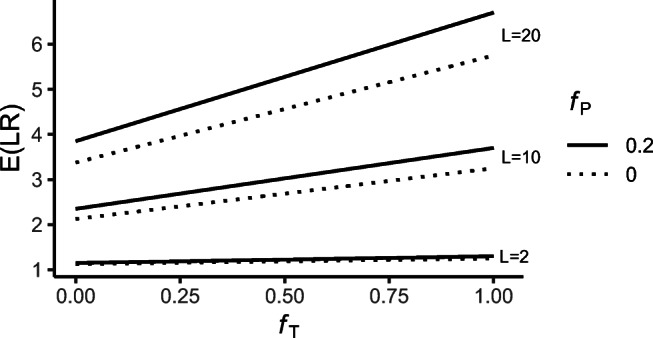
$E({\mathscr{L}}\mathcal {R})$ for the case in Fig. 6 as functions of background inbreeding level *f*_*T*_, for *f*_*P*_ = 0 (dashed line) and *f*_*P*_ = 0.2 (solid line), and *L* = 2, 10, and 20

In Fig. 7, the expectation of ${\mathscr{L}}\mathcal {R}$ is shown as a function of founder inbreeding *f*_*T*_ of the true half sibling pedigree, for *H*_*P*_ stating sibling pedigree with founder inbreeding ***f***_*P*_ = 0 and 0.2 (assuming *f*_1_ = *f*_2_), and *L* = 2, 10 and 20 alleles at a locus. For increasing values of *f*_*T*_, $E({\mathscr{L}}\mathcal {R})$ increases, for all values of ***f***_*P*_, and the evidence in favor of a sibling relationship becomes stronger.

**Fig. 8:**
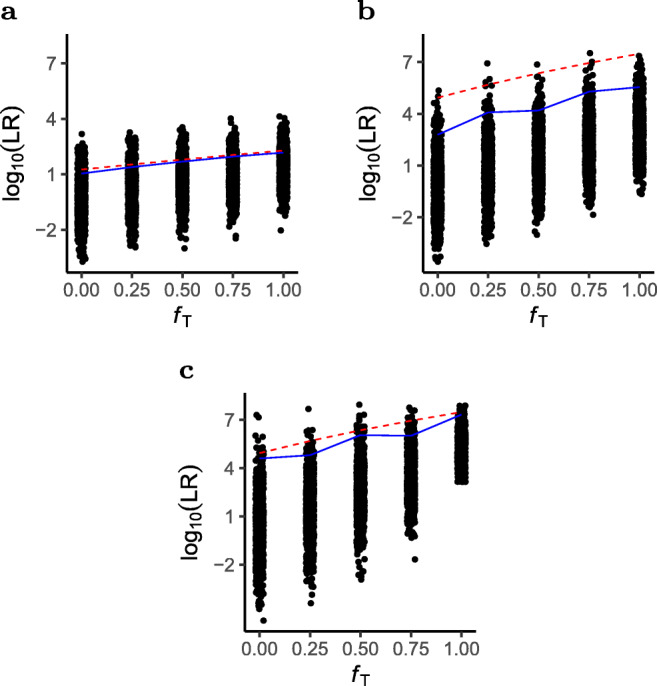
Simulations of *LR* for the case in Fig. 6. Each figure shows 1000 LR values, for five values of *f*_*T*_, each calculated from a simulation of a complete set of genotypes for 13 loci. Solid lines show mean of simulated *LR*. Dashed lines show $E({\mathscr{L}}\mathcal {R})$. **a** Loci with 3 alleles with frequencies 0.4, 0.3 and 0.3. **b** CODIS loci with realistic allele frequencies. **c** CODIS loci with uniform allele frequencies

Consider next the situation when *f*_1_ = *f*_2_ = 0. *H*_*P*_ then assumes a sibling relationship without inbred founders. Figure 8 shows $E({\mathscr{L}}\mathcal {R})$ (dashed line) and *LR* computations from 1000 sets of simulated data, as a function of *f*_*T*_. The solid line gives the mean value of the simulated *LR*. The expected LR increases slightly as founder inbreeding increases. For Fig. 8a this seems to fit well with the mean values of the *LR* s from simulated data. These simulation assumes 13 loci, each of 3 alleles with allele frequencies 0.4, 0.3 and 0.3. In Fig. 8b, on the other hand, there is a substantial difference between $E({\mathscr{L}}\mathcal {R})$ and the mean of the simulated *LR* s. These simulations use 13 CODIS markers with allele frequencies ranging from 0.0003 to 0.5378 (allele frequencies are available as a part of the R library InbredLR, see the “R implementation” section). Alleles with low frequencies will more seldom be present in the simulations. The expected LR only depends on the number of alleles at a locus, but because of the rare alleles, the simulations give in practice a lower number of alleles at these loci. The simulations in Fig. 8c use the same markers, but with uniform allele frequencies for alleles at a locus. The expectation of the LR is independent of the allele frequencies and is therefore not changed, but now the mean of the simulated *LR* s is closer to the expected value. Even though $E({\mathscr{L}}\mathcal {R})$ is independent of the allele frequencies, the variance is not, and small allele frequencies increase the variance.

Finally, we offer an approximation of the inflation in the expected LR due to background inbreeding. For simplicity, we assume *f*_1_ = *f*_2_ = 0 so that *H*_*P*_ states a normal sibling relationship. From Eq. 23 the expected LR is $\mu _{0}=\frac {1}{8}(L+7)$ if *f*_*T*_ = 0. On the other hand, if *f*_*T*_ > 0, the expected contribution to the LR is $\mu _{f} = \frac {1}{8}(L-1)f_{T}$. For *L* ≥ 5 we have $\frac {\mu _{f}}{\mu _{0}} \geq \frac {1}{3}f_{T}$, and it follows that

$$ (\mu_{0} + \mu_{f})^{N} = [(1 + \tfrac{\mu_{f}}{\mu_{0}})\mu_{0}]^{N} \geq (1 + \tfrac13fN) {\mu_{0}^{N}}. $$

A background inbreeding level of *f*_*T*_ will inflate the expected LR by at least $\frac 13f_{T}N$. For example, with *N* = 20 and *f*_*T*_ = 0.05, the inflation rate is greater than 33*%*.

### Paternity case with inbreeding

**Fig. 9:**
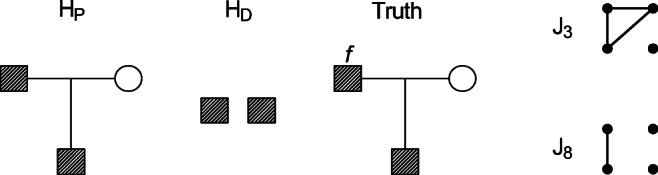
The hypotheses in a paternity case with inbreeding. To the far right are the Jacquard states with nonzero probability in the true relationship

Consider a paternity case with hypotheses as shown in Fig. 9. The alleged father is indeed the true father and has inbreeding coefficient *f*. We will analyze the consequences of ignoring the inbreeding in *H*_*P*_. The hypotheses are parameterized in the following way:

$$ \begin{array}{ll} H_{P}: \quad\boldsymbol{{\varDelta}}&=\boldsymbol{{\varDelta}}_{P} = (0, 0, 0, 0, 0, 0, 0, 1, 0) \\ H_{D}: \quad\boldsymbol{{\varDelta}}&=\boldsymbol{{\varDelta}}_{D} = (0, 0, 0, 0, 0, 0, 0, 0, 1) \\ Truth: \quad\boldsymbol{{\varDelta}}&=\boldsymbol{{\varDelta}}_{T}(f_{T}) \\ &=(0, 0, f_{T}, 0, 0, 0, 0, 1-f_{T}, 0). \end{array} $$

The expression for the expected LR simplifies considerably since most elements of **Δ**_*P*_ and **Δ**_*T*_(*f*_*T*_) are zero. Equation (15) gives

$$ E(\mathcal{L}\mathcal{R}) =\frac{L+1}{2}f_{T} +\frac{L+3}{4}(1-f_{T}), $$

and we see that $E({\mathscr{L}}\mathcal {R})$ increases linearly from (*L* + 3)/4 to (*L* + 1)/2 as *f*_*T*_ goes from 0 to 1.

Consider next the variance. For brevity, we define

24 $$ h(i,j,k)= E(\mathcal{L}\mathcal{R}_{J_{i},J_{k}}\cdot \mathcal{L}\mathcal{R}_{J_{j},J_{k}}).  $$

Note that *h*(*i*,*j*,*k*) is invariant under permutations of *i*,*j*,*k*. Equation (16) gives

$$ \begin{array}{ll} E(\mathcal{L}\mathcal{R}^{2})&={{\varDelta}^{T}_{3}} h(8,8,3)+{{\varDelta}^{T}_{8}} h(8,8,8)\\ &= f_{T} h(8,8,3)+(1-f_{T}) h(8,8,8). \end{array} $$

Slooten and Egeland [1] derived the term not involving inbreeding, i.e.,

$$ \begin{array}{ll} h(8,8,8) &=\frac{5L+3}{8}+\frac{s_{3}-L}{16}. \end{array} $$

To derive the remaining term we condition on the zygosity of the son. If he is homozygous a/a, the father must also be a/a (recall that we are conditioning on Jacquard state *J*_3_). Conversely, if the son is heterozygous a/b, the father is equally likely to be a/a or b/b. This gives

$$ \begin{array}{@{}rcl@{}} h(8,8,3) &=&\sum\limits_{a} {p_{a}^{2}} \frac{1}{p_{a}} \frac{1}{p_{a}}\\ &&+\sum\limits_{a< b} 2p_{a}p_{b}\left( \frac12 { (\frac{1}{2p_{a}})}^{2}+\frac12 {(\frac{1}{2p_{b}})}^{2} \right)\\ &=&  L + \frac14 \sum\limits_{a\neq b}\frac{p_{b}}{{p_{a}^{2}}} = \frac{3L+s_{3}}{4}. \end{array} $$

In summary,

25 $$ \begin{array}{@{}rcl@{}} &&\text{var}(\mathcal{L}\mathcal{R}) =\\ &&\frac{3L+s_{3}}{4}f_{T} + \left( \frac{5L+3}{8}+\frac{s_{3}-L}{16} \right)(1-f_{T}) \\ &&- \left( \frac{L+1}{2}f_{T} +\frac{L+3}{4}(1-f_{T})\right)^{2}. \end{array} $$

**Fig. 10:**
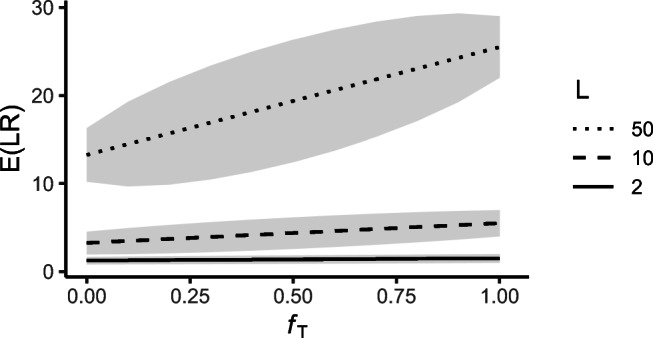
$E({\mathscr{L}}\mathcal {R})$ as a function of *f*_*T*_ in the paternity case in Fig. 9, for a single marker with *L* = 2, 10 and 50 alleles. Shaded area shows one standard deviation below and above $E({\mathscr{L}}\mathcal {R})$, for uniform allele frequencies

This is a concave function with respect to *f*_*T*_. Figure 10 shows $E({\mathscr{L}}\mathcal {R})$ and one standard deviation on each side as a function of founder inbreeding *f*_*T*_, for different number of alleles at a locus.

## Discussion

In testing theory, the formulation of hypotheses is crucial. Kinship problems, as considered in this paper, are no exception. The convention of kinship testing is to compare two specific relationships using the LR. In most applications other than kinship problems, the hypotheses together span many, if not all, alternatives. For instance, a common example is testing of HWE against *all* possible deviations from HWE. In forensic genetics, *H*_*P*_: “paternity” is typically tested only against *H*_*D*_: “unrelated,” not all other alternatives. For this reason, it becomes essential to study what happens when the truth is neither of these hypotheses.

A pairwise non-inbred relationship can be presented by a point in the IBD triangle (see Fig. 2), or in general by the Jacquard coefficients (see Fig. 1). We have presented two ways of expressing the hypotheses and the true relationship; (i) through the Jacquard coefficients, and (ii) background relatedness or founder inbreeding. These approaches let us investigate the LR for a continuous range of relationships and values of background relatedness. In both cases, the impact on the LR has been studied by deriving exact expressions for its mean and variance. In the latter case, the required formula follows rather directly by extending results in [1] and [2]. Explicit formulas for the expected LR has been derived for several sets of relationships. In the case of Jacquard coefficients, the explicit formulas are complicated to derive, and they depend on allele frequencies. An exact expression is given also for the variance. However, as the variance depends on allele frequencies, simple closed formulas can only be derived in special cases. For general applications we rely instead on the exact numerical implementation freely available in the R library InbredLR accompanying this paper.

Equipped with the results of this paper, we can address the following question when presented with a standard LR comparing two completely specified hypotheses *H*_*P*_ and *H*_*D*_: What if the true relationship between the individuals is not as stated by *H*_*P*_? Or this slightly different question: What if the true relationship is restricted to some particular region of the IBD triangle. Obviously, the LR can be re-evaluated to reflect the new specifications. However, the exact expressions for expectation and variance of the LR can in some cases directly allow for statements valid for a continuous range of alternatives. For instance, regions obtained by varying founder inbreeding have been displayed in Fig. 2. Assume a LR has been reported in a paternity case and that inbreeding in the father has been ignored. It is then useful to know that accounting for inbreeding would imply increase in the expected LR. This finding could be essential as there may not be data available to estimate the inbreeding coefficient for the father. Hence, exact LR calculation is not feasible.

Because the definition of “common ancestor” sometimes differs, there is a slight difference in the definition of IBD in the literature. The paper [16] gives three definitions of IBD: ancient IBD, recent IBD, and familial IBD. Our definition of IBD goes in the category of familial IBD, where “common ancestor” is restricted to a given pedigree.

The conventional approach to background relatedness in forensics is the so called theta (*𝜃*) correction [12]. Typical values are *𝜃* ∈ (0.01,0.03). The *𝜃* parameter applies on a population level. The genotype probabilities of all founders in the pedigree are modified compared with what HWE would give. Our approach does not model relatedness between founders, but offers a richer model of inbreeding, since individual inbreeding coefficients can be specified for each founder.

Several authors (see, e.g., [2] and the references therein) have discussed reporting the logarithm of the LR rather than the LR. Nice expressions like the ones presented for the expectation and the variance are then no longer available. In most cases, the LR is reported on the original scale. In some circumstances, as for paternity cases, the LR may be 0, and then, the logarithm is not defined. Many papers including [17] study the distribution of $\mathcal {Z}=\log ({\mathscr{L}}\mathcal {R})$ by simulation. Equipped with the exact expressions of this paper, $\mathcal {Z}$ could be analyzed without resorting to simulation, since the mean and variance of $\mathcal {Z}$ can be derived from the counterparts for the LR. However, if some allele frequencies are close to 0, $\mathcal {Z}$ is not well approximated by a normal distribution for a realistic number of markers. The reason for this is the large variance when allele frequencies are small. For instance, (25) shows an example where the expression for the variance include terms of the form 1/*p*_*a*_ and these become large whenever the allele frequency *p*_*a*_ is small. A similar problem related to small allele frequencies is discussed in the result section. This demonstrates that the center of the $\log ({\mathscr{L}}\mathcal {R})$ distribution, calculated from the expectation of ${\mathscr{L}}\mathcal {R}$, can be inaccurate. However, this criticism applies to the use of ${\mathscr{L}}\mathcal {R}$ instead of $\log ({\mathscr{L}}\mathcal {R})$ in general, and not specifically to the expectations. We maintain that results like the ones presented for the expectation and variance have considerable theoretical interest, but should be used with caution in practice.

This paper has mainly addressed the likelihood ratio and its properties. The exclusion probability (EP), the probability that genotypes will be incompatible with a claimed relationship, is also an important statistic. The impact of founder inbreeding on EP is discussed in [3].

Figure 4 illustrates a case where the true inbred relationship is not known, and Fig. 5 shows the corresponding expected LR for a single marker. Increasing the number of markers will, in this paternity case, increase the inflation of the expected LR. This means that adding more markers to the LR computation will not solve the problem. In general, with a sufficient number of markers, the Jacquard, IBD, or inbreeding coefficients can be estimated accurately, and the true relationship detected. If such additional marker data is not available, the impact of inbreeding can be studied as exemplified by a paternity case with unknown inbreeding earlier in the discussion and as illustrated in, e.g., Fig. 5. As addressed in the “Introduction” section, different scenarios can be investigated and LR results can be evaluated in light of the analyses of these scenarios.

The present paper does not consider linked markers. For independent loci, the inbreeding coefficients contain sufficient information to compute the Jacquard coefficients needed in our formulas for LR. While a similar approach is conceivable also for linked markers, this would involve multi-locus coefficients, which is outside the scope of this work.

## Appendix: Expectation and variance of LR

Below we derive the expressions for the expectation and variance of ${\mathscr{L}}\mathcal {R}$ in the general pairwise case. Let *J*_*i*_ denote Jacquard state *i* and ${{\varDelta }_{i}^{P}}$ and ${{\varDelta }_{i}^{T}}$ the probabilities of *J*_*i*_ according to the relationship stated by *H*_*P*_ and the true relationship respectively. ${\mathscr{L}}\mathcal {R}_{\boldsymbol {{\varDelta }}_{P},\boldsymbol {{\varDelta }}_{T}}$ is then defined as the likelihood ratio comparing *H*_*P*_:**Δ**_*P*_ with *H*_*D*_:**Δ**_*D*_ when the marker data comes from the relationship **Δ**_*T*_. Similarly, ${\mathscr{L}}\mathcal {R}_{J_{i},J_{j}}$ denotes the likelihood ratio comparing Jacquard state *J*_*i*_ with unrelated, i.e., *J*_9_ when the marker data are generated by *J*_*j*_.

Equation (15) follows by combining (1), (14), and (4)

26 $$ \begin{array}{ll} E(\mathcal{L}\mathcal{R})&=\sum\limits_{G}\left( \sum\limits_{j=1}^{9}{{\varDelta}_{j}^{T}}P(G\mid J_{j})\sum\limits_{i=1}^{9}{{\varDelta}_{j}^{P}}\frac{P(G\mid J_{i})}{P(G\mid J_{9})}\right)\\ & = \sum\limits_{i=1}^{9}\sum\limits_{j=1}^{9}{{\varDelta}_{i}^{P}}{{\varDelta}_{j}^{T}}\left( \sum\limits_{G}\frac{P(G\mid J_{i})}{P(G\mid J_{9})}P(G \mid J_{j})\right)\\ &=\sum\limits_{i=1}^{9}\sum\limits_{j=1}^{9}{{\varDelta}_{i}^{P}}{{\varDelta}_{j}^{T}}E\left( \mathcal{L}\mathcal{R}_{J_{i},J_{j}}\right)\\ &= \boldsymbol{{\varDelta}}_{P}B_{9}{\boldsymbol{{\varDelta}}_{T}^{t}}. \end{array} $$

In the case of no inbreeding, i.e., *Δ*_1_ = ⋯ = *Δ*_6_ = 0, the above expression reduces to (5). The part of the 9 × 9 matrix *B*_9_ corresponding to (*J*_7_,*J*_8_,*J*_9_) coincides with the matrix given in Eq. 6. Since $E({\mathscr{L}}\mathcal {R}_{J_{i},J_{j}})=E({\mathscr{L}}\mathcal {R}_{J_{j},J_{i}})$, *B*_9_ is symmetric. The elements of *B*_9_ are found by direct calculation. For instance, entry (1,1) equals

$$ E(\mathcal{L}\mathcal{R}_{J_{1},J_{1}}) = \sum\limits_{a}\frac{p_{a}}{{p_{a}^{4}}}p_{a} =\sum\limits_{a} \frac{1}{{p_{a}^{2}}}. $$

Since the expectation has been calculated, to derive the variance it remains only to find

$$ \begin{array}{@{}rcl@{}} &&E(\mathcal{L}\mathcal{R}^{2})\\ &=& \sum\limits_{G} \left( \sum\limits_{k=1}^{9}{{\varDelta}_{k}^{T}}P(G\mid J_{k})\sum\limits_{i=1}^{9}{{\varDelta}_{i}^{P}}\frac{P(G\mid J_{i})}{P(G\mid J_{9})}\right.\\ &&\left.\sum\limits_{j=1}^{9}{{\varDelta}_{j}^{P}}\frac{P(G \mid J_{j})}{P(G\mid J_{9})}\right)\\ &=&\sum\limits_{i=1}^{9} \sum\limits_{j=1}^{9} \sum\limits_{k=1}^{9}{{\varDelta}_{i}^{P}} {{\varDelta}_{j}^{P}} {{\varDelta}_{k}^{T}}E\left( \mathcal{L}\mathcal{R}_{J_{j},J_{i}}\mathcal{L}\mathcal{R}_{J_{k},J_{i}}\right)\\ &=&\sum\limits_{i=1}^{9} {{\varDelta}^{P}_{i}} \boldsymbol{{\varDelta}}_{P} B_{i} {\boldsymbol{{\varDelta}}_{T}^{t}}. \end{array} $$

The matrices *B*_1_,…,*B*_9_ are symmetric 9 × 9 matrices. The simplest of these matrices is *B*_9_, given in Table 4. In general, *B*_*i*_ consists of the elements $\{E({\mathscr{L}}\mathcal {R}_{J_{j},J_{i}}{\mathscr{L}}\mathcal {R}_{J_{k},J_{i}})\}_{j,k=1,\ldots , 9}$. The values for *i*,*j*,*k* = 7, 8, 9 have been provided in the “Review of previous results” section. Entry (*j*,*k*) of *B*_*i*_ is

27 $$ \sum\limits_{G} \frac{P(G\mid J_{j})}{P(G\mid J_{9})}\frac{P(G\mid J_{k})}{P(G\mid J_{9})}P(G\mid J_{i}). $$

All matrices can in principle be found from the above expression, but exact calculations by hand become unpractical and exact numerical calculation is more reasonable.
